# A Brake-Based Overground Gait Rehabilitation Device for Altering Propulsion Impulse Symmetry

**DOI:** 10.3390/s21196617

**Published:** 2021-10-05

**Authors:** Siyao Hu, Krista Fjeld, Erin V. Vasudevan, Katherine J. Kuchenbecker

**Affiliations:** 1 Department of Mechanical Engineering & Applied Mechanics, GRASP Laboratory, University of Pennsylvania, Philadelphia, PA 19104, USA; 2 Haptic Intelligence Department, Max Planck Institute for Intelligent Systems, 70569 Stuttgart, Germany; kjk@is.mpg.de; 3 Department of Physical Therapy, SUNY Stony Brook University, Stony Brook, NY 11794, USA; krista.fjeld@stonybrook.edu; 4 Department of Neurobiology and Behavior, SUNY Stony Brook University, Stony Brook, NY 11794, USA; erin.vasudevan@stonybrook.edu

**Keywords:** gait rehabilitation, leg propulsion, stance-phase resistance, overground walking

## Abstract

This paper introduces a new device for gait rehabilitation, the gait propulsion trainer (GPT). It consists of two main components (a stationary device and a wearable system) that work together to apply periodic stance-phase resistance as the user walks overground. The stationary device provides the resistance forces via a cable that tethers the user’s pelvis to a magnetic-particle brake. The wearable system detects gait events via foot switches to control the timing of the resistance forces. A hardware verification test confirmed that the GPT functions as intended. We conducted a pilot study in which one healthy adult and one stroke survivor walked with the GPT with increasing resistance levels. As hypothesized, the periodic stance-phase resistance caused the healthy participant to walk asymmetrically, with greatly reduced propulsion impulse symmetry; as GPT resistance increased, the walking speed also decreased, and the propulsion impulse appeared to increase for both legs. In contrast, the stroke participant responded to GPT resistance by walking faster and more symmetrically in terms of both propulsion impulse and step length. Thus, this paper shows promising results of short-term training with the GPT, and more studies will follow to explore its long-term effects on hemiparetic gait.

## 1. Introduction

Mobility impairments are a frequent cause of stroke-related disability: among ischemic stroke survivors 65 years and older, half reported hemiparesis (weakness on one side) persisting six months post-stroke, and 30% were unable to walk without assistance [1]. Difficulty with functional movements like gait can lead to physical deconditioning post-stroke, which contributes to poor cardiovascular fitness, muscular atrophy, and metabolic syndrome [2]. These effects can increase the risk of a second stroke or cardiovascular event [2]. Motor impairments are also associated with negative psychological and social outcomes, such as depression and withdrawal [3].

There are several training strategies aimed at increasing walking function after stroke [4,5]. However, many strategies appear to deliver comparable outcomes and may not result in meaningful gains in walking speed [6]. It is possible that treatments correcting a specific functionally limiting aspect of gait may be more effective than more global treatments that aim to correct a variety of functional deficits during walking practice (e.g., body-weight-supported treadmill stepping). Recently, *propulsion asymmetry after stroke* was identified as a functionally limiting gait deficit that is correlated with walking speed [7,8,9,10]. A stroke that results in hemiparesis is often accompanied by propulsion asymmetry, which occurs when the more affected (i.e., paretic) leg is unable to generate propulsion forces that match those on the less affected (i.e., non-paretic) leg. Propulsion asymmetry between the two legs is negatively correlated with walking speed and functional mobility; conversely, improvements in propulsion asymmetry with training are associated with improvements in walking speed [8,11,12]. These findings suggest that targeting propulsion asymmetry during gait training is a promising approach for improving functional mobility after stroke, although we note that the reported speed increase is typically in post-intervention conditions and not during intervention [8,10]. For a person with hemiparetic gait, propulsion must be increased in the paretic leg to match the propulsion of the contralateral leg, which may reduce, increase, or stay constant. However, because walking is a bilateral activity, it can be challenging to focus propulsion training on only one leg.

One way to accomplish unilateral propulsion training is through functional electrical stimulation (FES), which administers an electric pulse to stimulate muscle contraction at appropriate times during the gait cycle. Training walking with FES applied to ankle musculature results in increased paretic leg propulsion, reduced gait asymmetry, and increased walking speed after stroke [8,11]. However, one possible disadvantage of using FES is that the pattern of muscle fiber recruitment is non-specific [13] and dissimilar to the pattern of recruitment found naturally in gait [14]. Gait rehabilitation that involves mechanical assistance or resistance training may have advantages over electrical training, due to the natural order of muscle recruitment, particularly for people who are ambulatory and have some ability to voluntarily recruit leg muscles. Additionally, FES may be contraindicated in people with pacemakers and skin lesions; thus, it is not an appropriate treatment for everyone.

Robotic devices can potentially be used for unilateral mechanical gait training because they can be configured for various output modes and different levels of interaction with the user [15]. For example, the LOwer extremity Powered ExoSkeleton (LOPES) allows both ‘patient-in-charge’ (e.g., for the unimpaired limb) and ‘robot-in-charge’ (e.g., for the impaired limb) modes during training [16]. In most existing approaches, robotic devices provide assistance to users during training, which allows the patient to practice motions that self-generated effort cannot achieve. However, too much assistance may also reduce physical effort during motor training [17] and may encourage slacking [15]. In addition, the assistance provided by robotic devices typically relies on a desired trajectory, but commonly used normative movements, such as minimum-jerk trajectories and trajectories recorded from unimpaired volunteers, have not been proved to maximize training effect [15]. Although it is possible to infer the desired motion of the paretic limb from the unimpaired leg for gait training [18], the stability of this approach for stroke patients remains to be determined.

In contrast, robotic resistance devices have been less extensively investigated for unilateral gait training. A few studies have found that non-robotic resistance exercises that require higher effort from the impaired limb could indeed help people with stroke improve their motor function [19,20,21]. One study used an exoskeletal robotic gait device to apply resistance and found that velocity-dependent moments against hip and knee movements during the swing phase in people with spinal cord injury resulted in enhanced knee flexion after the resistance was removed [22]. Another common training scheme is to apply resistance forces at the ankle via a cable. One research team hung a weight at the other end of the cable via a pulley system; they found that people with hemiparesis could adapt both spatial and temporal aspects of their gait under this force, although the learning rate was reduced in the late adaptation phase [23]. Note that this mechanism applies the force from the weight during all gait phases, including stance. By replacing the weight with motors in front of and behind the user, one can apply swing-phase resistance and/or assistance at selected times [24]. Gait asymmetry in people with stroke improved more following a single period of walking with swing-phase resistance applied at the ankle, compared to swing-phase assistance [25]. However, another study using the same device did not find a significant difference in walking speeds between people receiving 6 weeks of assistance or resistance training [26], suggesting that there is much room to explore the best resistance training schemes and how they can help with motor recovery.

Recently, novel training schemes were introduced to target stance-phase propulsion. In one approach, a leg exoskeleton was used to apply torque pulses to the participant’s hip and knee joints during stance in both single- and repeated-pulse walking experiments [27]. Depending on the timing of the pulses, the effects on hip extension and normalized propulsion impulse metrics could be negative or positive, although the after-effects were generally positive, especially with early stance flexion torque [27]. In another approach based on split-belt treadmill training, the belt of the trailing limb was accelerated to induce greater propulsive force and larger extension of the trailing limb during push-off [28]. The self-chosen gait speed of people experiencing this acceleration intervention significantly increased after training, compared to the control group [28].

These examples help motivate our research into a new approach for robotic stance-phase resistance training, which we postulate could improve propulsion symmetry and thus walking function. To the best of our knowledge, periodic resistance training schemes like our proposed method have not been previously studied. This paper describes the Gait Propulsion Trainer (GPT), a novel device that produces periodic stance-phase resistance during overground walking via a rotary magnetic-particle brake, a cable, a waist-worn harness, and event switches. In addition to describing the design, we verify the hardware and report a pilot study in which one healthy adult and one stroke survivor each walked with increasing periodic resistance from the GPT. The results show that our approach induces greater propulsion impulse contribution from the resisted side for the healthy participant and from the paretic side for the stroke participant, as hypothesized.

## 2. Materials and Methods

### 2.1. Design Motivation

At the moment, the most effective resistance training for gait rehabilitation is undetermined. Thus, we aim to design a robotic device that can apply programmable stance-phase resistance targeting unilateral propulsion during walking, while maintaining safety. This device will then serve as the platform for following studies that determine the most effective control scheme for resistance gait training.

First, the requirement of stance-phase resistance eliminates the common approach of applying robotic disturbance at the ankle. Since the foot is stationary during the stance phase, robotic disturbance of the ankle would do little to no mechanical work, and any positive work could destabilize the user. Instead, we seek to apply the resistance at the pelvis, which is propelled to advance forward in space by the leg in stance phase. We postulate that such resistance could lead to increased propulsion from the resisted leg and improved propulsion symmetry, similar to how step-length symmetry was shown to improve with resistance training [25].

Second, maintaining safety means that any positive work done on the user (typically by motors in robotic devices) must be extensively regulated, or a protective mechanism must be in place in case of instability (e.g., the harness in [25] was used for safety but not for body weight support). Actuators that dissipate energy, such as brakes, generate resistance forces by acting against the user’s motions, making them a good option for safe resistance intervention. Since we aim to apply large resistance forces, we propose to tether the user’s pelvis to a stationary brake via a cable so that resistance forces can be applied by the brake as the user walks away. A similar mechanism was validated for haptic feedback to the hand in our prior work [29]. We focus our investigation on overground training because it offers improvements of walking function that are comparable to or greater than training on a treadmill without the cost and space that a treadmill requires [30,31,32].

Third, for unilateral intervention during the stance phase, our preliminary control scheme is to turn on the resistance force when one leg is in single-limb support; that is, the resistance starts when only the resisted (i.e., ipsilateral) leg is in contact with the ground and the contralateral leg is in swing phase. It then turns off when the resisted leg lifts off the ground (‘toe-off’) and initiates the swing phase, which means that the resistance force is still applied during double support. We choose to apply resistance forces during contralateral-leading double support in our preliminary control scheme because the resisted leg may still be generating propulsion forces at this time. In people without neurological damage, the duration of the period starting at the onset of single limb support to toe-off on one side is a little less than 50% of the gait cycle [33], or about 0.4–0.5 s, depending on how fast the person is walking. In people with stroke, this period can be variable: it may be longer than 0.5 s in some people because they walk slower, and it may be shorter in others because people with stroke spend less time in single-limb stance on their weaker side. Regardless, accurate and fast gait phase detection is required. Given that we allow overground walking and apply resistance only to the pelvis, an orthosis may be cumbersome and introduce erroneous interaction with the user, while drastically increasing the cost of the system. In contrast, a lightweight and easy-to-use wearable device with wireless data transmission would be ideal for gait-phase detection.

### 2.2. Prototype Design

**Figure 1:**
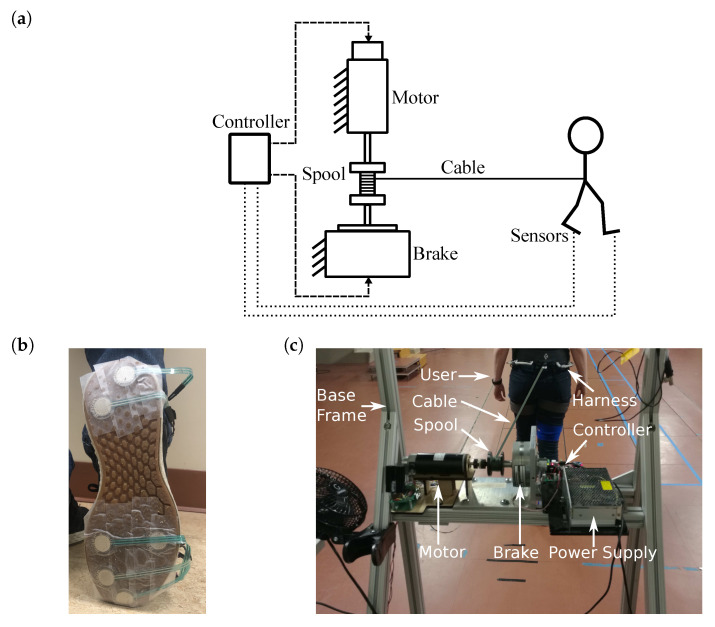
Schematic and a prototype of the proposed GPT. (**a**) GPT schematic. A person is tethered to a spool, which is connected to a motor and a brake. Dashed lines represent wired connections, and dotted lines show wireless data transmission. (**b**) Event switches on a shoe sole. (**c**) The stationary device. The cable is digitally enhanced to increase visibility.

The overall design of the GPT is shown in Figure 1a. A trial with GPT training starts with the user standing in front of the GPT and ends when the user can no longer walk forward (for example, the person reaches the end of the walkway or the other side of the room, or the person needs to stop and rest). As the user walks during each trial, the cable unwinds, and periodic resistance forces are applied. After each trial, the cable is reeled in, and the user returns to the GPT to start another trial.

#### 2.2.1. Sensing System and Wearable Equipment

The sensing system uses twelve MA-153 event switches (Motion Lab Systems, Los Angeles, CA, USA) to determine the user’s gait phase. Each event switch reads as ‘on’ when there is contact on its surface, and ‘off’ for no contact. Six event switches are attached to the bottom of each of the user’s shoes: four underneath the metatarsal bones, and two underneath the heel, as shown in Figure 1b. This setup is similar to the gait phase detection system in [34], but without a gyroscope and with two event switches wired in parallel instead of each force-sensing resistor for improved reliability. An ATmega32U4-based microcontroller reads the outputs from the event switches and wirelessly transmits them to the stationary device at 100 Hz using an XBee module (Digi International, Hopkins, MN, USA). The microcontroller and wires are attached to the participant’s thigh, using soft straps.

The user also wears a climbing harness (Black Diamond Equipment, Holladay, UT, USA) below his or her waist. The back of the harness can be attached to a cable, which we use to tether the user to the stationary device and apply the resistance force to the pelvis. Fishing line (PowerPro, Irvine, CA, USA) is used as the cable, because of its low weight, high strength, and high flexibility.

#### 2.2.2. Stationary Device

As shown in Figure 1c, the stationary device consists of an aluminum frame to which the other components are attached. Its height is adjustable to accommodate users of different heights. A 24 V S90MPA-B45D75S rotary magnetic-particle brake (Stock Drive Products/Sterling Instrument, Hicksville, NY, USA) is fixed at the top center of the frame. The maximum output torque of this brake is 6.78 Nm, and its inherent friction (minimum output) is 0.11 Nm. The brake is driven by a linear current amplifier. A 3D-printed spool with a diameter of 3.18 cm is mounted on the shaft of the brake, and the cable that tethers the waist-worn harness is reeled on the spool.

A 30 V DCM50207 DC motor (Leadshine, Shenzhen, China) with a 1000-line quadrature encoder (4000 counts per revolution) is connected to the spool via a flexible shaft coupler on the opposite side of the magnetic-particle brake, as shown in Figure 1c. The maximum continuous output torque of this motor is 0.35 Nm. A similar current amplifier is used to control the output torque of the motor.

An ATmega32U4-based microcontroller wirelessly receives event switch readings from the sensing system and determines the user’s gait events on each side, including stance, toe-off (stance-to-swing transition), swing, and heel-strike (swing-to-stance transition), as defined in [34]. The outputs of the actuators are subsequently determined by the gait phase, as detailed in Section 2.2.4. The microcontroller also reads the motor encoder values. During experiments, the microcontroller sends sensor readings and control signals to a PC for recording at approximately 30 Hz.

#### 2.2.3. Actuator Control

When the person walks away from the stationary device, we use the motor to generate an assisting torque that partially cancels the inherent friction of the rotary brake, i.e., the motor applies a torque in the direction of cable unwinding. This friction compensation reduces the resistance force applied on the user during unresisted limb stance to emphasize unilateral training. With the GPT prototype, we increase the motor torque from zero until a magnitude such that when the brake is not activated, the shaft starts turning with minimal pulling on the cable and immediately stops turning once the user stops walking. We achieve a friction reduction of 0.06 Nm (about 54% of the brake’s inherent friction).

When the user reaches the end of a trial and the GPT needs to reset, we detach the cable from the harness and then use the motor to turn in the direction that reels in the cable. The harness is then attached again, and a new trial can begin.

The rotary brake is used to generate a pelvic resistance force during training. As the participant walks away from the stationary device, the cable unreels and causes the spool and the brake shaft to rotate. When the brake is activated, we have the following:

(1) $$\begin{array}{c} T=\frac{2\tau }{d}, \end{array}$$

where *T* is the cable tension, τ is the braking torque, and *d* is the spool diameter. We use *T* as the nominal value for the resistance force on the user.

**Figure 2:**
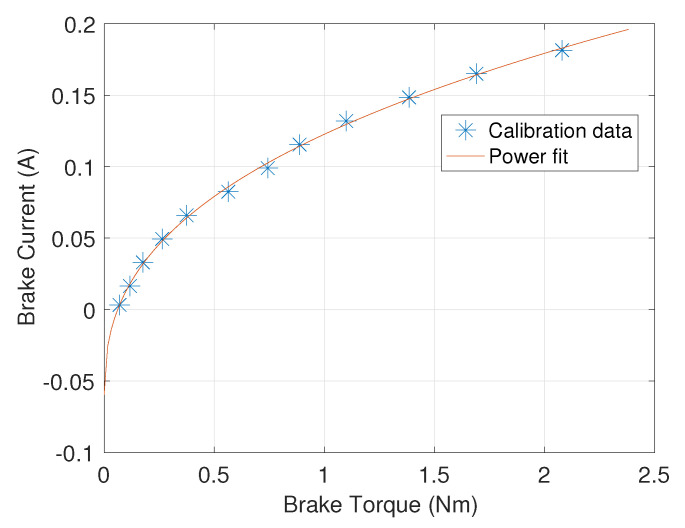
Torque-current relationship of the brake.

To calibrate the brake’s current/torque relationship, an iLoad Mini force sensor (LoadStar Sensors, Fremont, CA, USA) rated up to 222.41 N is tethered to the loose end of the cable. An operator holds the force sensor and walks away from the GPT while the motor is actuated in the forward direction with the assisting torque, and selected current levels are sent through the brake. The mean value of the force data that is recorded while the shaft rotates is converted to torque, using Equation (1), and is used to calibrate the brake, as shown in Figure 2. We obtain the following power model from the data, using the curve fitting tool in MATLAB (Mathworks, Natick, MA, USA):

(2) $$\begin{array}{c} \begin{array}{cc} i= & -0.05951\;A+\left(0.01538\;A/\sqrt{\mathrm{Nm}}\right)\tau^{\frac{1}{2}}\\ & +\left(0.2644\;A/\sqrt[{3}]{\mathrm{Nm}}\right)\tau^{\frac{1}{3}}-\left(0.09759\;A/\sqrt[{4}]{\mathrm{Nm}}\right)\tau^{\frac{1}{4}} \end{array} \end{array}$$

where *i* is the current through the brake, and τ is the braking torque. Note that the power fit gives negative current values when desired torque outputs are close to zero because the inherent friction of the brake is not entirely canceled by the motor. A negative current generates the same resistance force as a positive current in such a brake. Therefore, we put a lower bound of 0 A on the brake current command.

**Figure 3:**
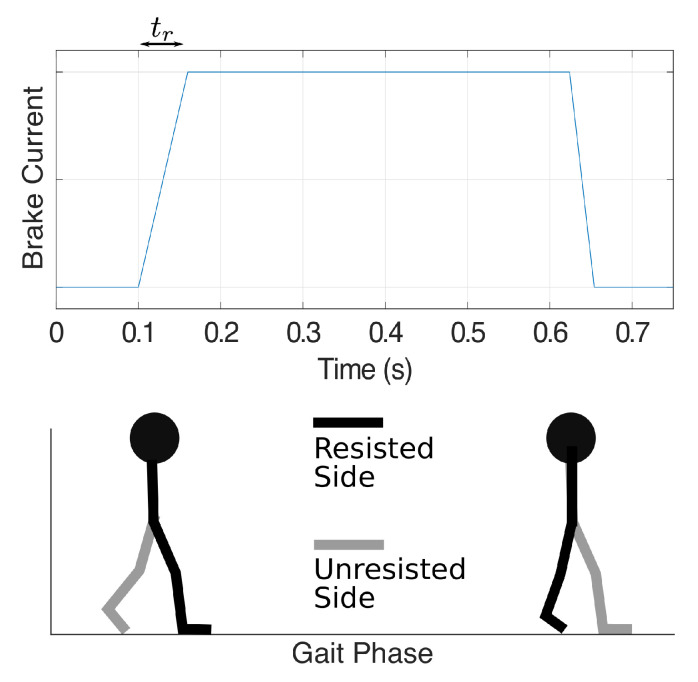
Illustration of the brake current in an example step with corresponding gait phases as the user walks, where $t_{r}$ represents the ramp-up time when the brake is first actuated.

When the control signal for the brake driver increases instantly from zero to a high value, the resultant torque tends to behave like static friction: an initial load torque higher than the steady-state torque is needed to start the shaft rotation. To avoid this unwanted effect when the brake is first actuated, we set the brake current to linearly rise from zero to the desired level within a specified time interval, as shown in the top panel in Figure 3. Our experiments use a ramp-up time of $t_{r}=60\;ms$ to achieve smooth force onset and allow force application during a high proportion (∼87%) of the stance phase. We expect the optimal ramp-up time to depend on the non-ideal properties of the brake itself, which might change over long periods of time, so this value can be adjusted.

#### 2.2.4. GPT Controller

**Figure 4:**
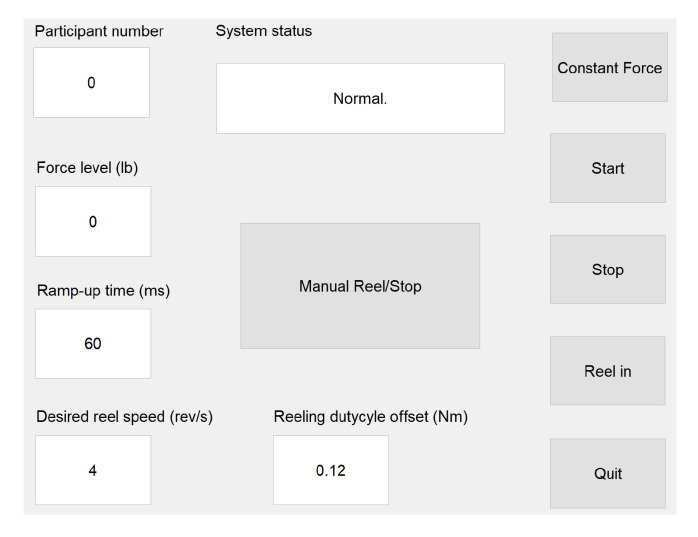
Matlab GUI. When the program starts, the operator enters the participant number, the desired force level, and other relevant parameters, if necessary. The operator then controls the GPT via the push buttons and monitors the system status.

We implement a state-machine controller for the GPT to determine the desired output for the two actuators. An operator controls the system via a graphical user interface (GUI) in Matlab running on a PC, as shown in Figure 4. The participant number, desired resistance force level, ramp-up time ($t_{r}$), reeling speed, and reeling duty cycle offset are specified in the text input fields. Note that the reeling parameters can be adjusted to change the cable reeling speed, but they have no effect on training because reeling takes place between trials. The ‘System status’ field shows pre-defined messages that indicate the operation modes of the GPT and error messages, if any. Buttons in the GUI correspond to some of the transition signals in the state-machine controller.

**Figure 5:**
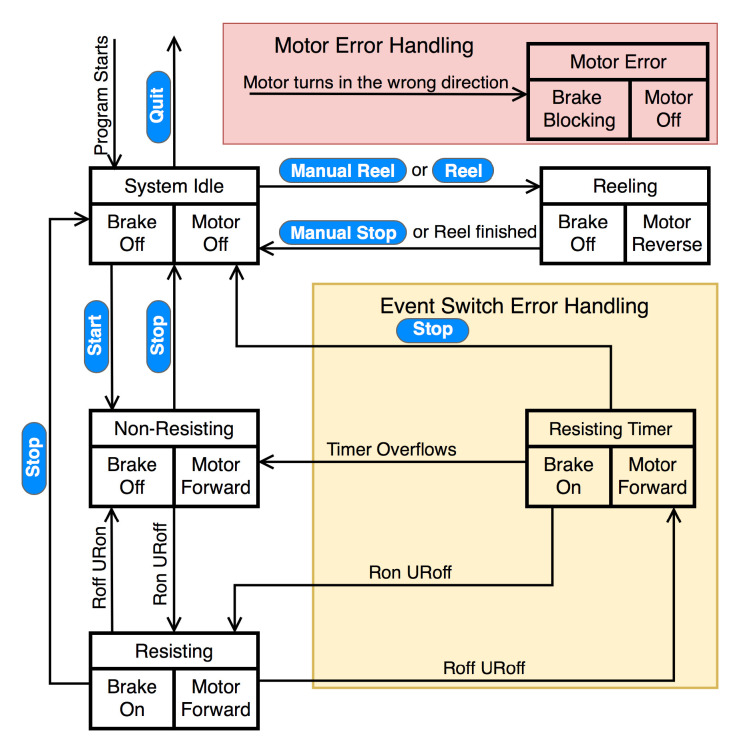
State-machine controller for the GPT. Each state has a name (e.g., ‘Non-Resisting’) and descriptions of the actuators (e.g., ‘Brake Off’ and ‘Motor Forward’). ‘Ron’ represents when any of the event switches on the resisted side reads ‘on’, i.e., when the resisted leg is in the stance phase. ‘Roff’ represents when all these event switches read ‘off’, i.e., when the resisted leg is in the swing phase. ‘URon’ and ‘URoff’ similarly represent the unresisted side.

As shown in Figure 5, when the program starts, the GPT enters the ‘System Idle’ state, where both the brake and the motor are off. After the operator clicks the ‘Start’ button, the GPT zeros the motor encoder reading and enters the ‘Non-Resisting’ state, where the brake is off. In this state, the motor is activated in the forward direction with the assisting torque, so the inherent friction of the rotary brake is partially canceled; the motor remains activated in the forward direction until the ‘Stop’ button is clicked.

As discussed in Section 2.1, our preliminary training scheme aims to turn on the resistance force at the beginning of the resisted leg’s single-support phase and turn off the force when the resisted foot leaves the ground. Thus, when the resisted foot is in the stance phase and the unresisted foot is in the swing phase (single-limb stance; arrow marked ‘Ron URoff’), the GPT enters the ‘Resisting’ state, where the brake is activated to apply the desired resistance force to the user. When the unresisted leg is in stance phase and the resisted foot leaves the ground (arrow marked ‘Roff URon’), the GPT returns to the ‘Non-Resisting’ state. The desired resistance forces and the corresponding gait phases are illustrated in Figure 3 for transitions between the ‘Non-Resisting’ and ‘Resisting’ states; only toe-off events are needed to control the resistance forces in the current training scheme.

After the operator clicks the ‘Stop’ button in the GUI, the controller returns to the ‘System Idle’ state. All data collected during the walking trial, including time-stamped event switch and encoder readings and the control signal for the rotary brake, are then saved to the PC. After the user is detached from the cable, the operator can click the ‘Reel’ button for the GPT to enter the ‘Reeling’ state, where the rotary brake remains off and the motor automatically rotates the spool in the reverse direction until the loose cable is fully reeled. At the same time, the user can walk or sit in a wheelchair and come back to the starting point near the stationary device.

To ensure safety, we add error-handling states that determine how the GPT should behave if a sensor or actuator misbehaves. One possible type of error is with the event switches under the user’s shoes. Under normal conditions, the event switches should read as on and off periodically, indicating the gait phases as the user walks forward. However, if the event switches are not securely attached to the correct locations under the shoes, there may be time periods where all event switches read as no contact, suggesting that neither of the user’s feet is in contact with the ground. However, this scenario should never happen because the user should be walking and not running or jumping when training with the GPT. Thus, to avoid sudden changes in the brake force when the event switches misread, especially when no event switches are pressed during the ‘Resisting’ state (arrow marked ‘Roff URoff’), an extra ‘Resisting Timer’ state imposes a short delay before turning off the brake resistance force, as shown in the ‘Event Switch Error Handling’ box in Figure 5. Note that this error handling state and its transitions are enclosed in the yellow region, indicating that this error is recoverable and the GPT can continue normal functioning if all event switches still read as no contact or if expected event switch readings are acquired within the short delay.

Secondly, a rare but potentially dangerous failure of the GPT is when the DC motor turns unexpectedly, particularly in the reeling direction, which may be caused by incorrect motor installation or driver breakdown. Readings from the motor’s quadrature encoder are used to detect this failure, and the ‘Motor Error Handling’ box shown in Figure 5 enables the GPT to react quickly and ensure that the user is never actively pulled: when triggered, the GPT turns off the motor output immediately and activates the magnetic brake to generate a large torque and completely block the shaft from turning. Note that this error handling component is enclosed in the light red region to represent that the ‘Motor Error’ state has the highest priority in the state machine and can be entered from every other state. The operator must completely turn off the system once the GPT enters the ‘Motor Error’ state.

### 2.3. Evaluation Methods

In order to evaluate the performance of the GPT prototype, we performed two tests. In the *hardware verification test*, we validated the objective GPT characteristics, including resistance force control and the time delay between corresponding gait events and GPT resistance actuation. The second test was a *pilot study*, where one healthy participant and one stroke survivor tested the device, and the propulsion forces were measured by force plates. Both subjects consented to experimental procedures, which were approved by the Institutional Review Board at Stony Brook University in accordance with the Declaration of Helsinki.

**Figure 6:**
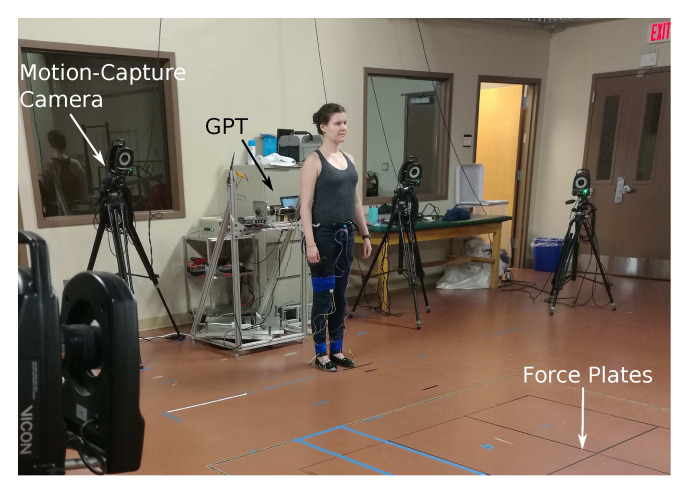
A user in front of the GPT ready to start training. She will walk over the in-ground force plates while feeling periodic resistance through the harness, and her kinematics will be recorded by the motion-capture system. The force plates are covered during actual training, so users do not adjust their step locations unnaturally.

In these two tests, the GPT was placed at one end of a 10 m overground walkway that has four embedded in-ground force plates (AMTI Force and Motion, Watertown, MA, USA). As shown in Figure 6, the motion of the participant was measured using an optical motion-analysis system (VICON, Denver, CO, USA).

#### 2.3.1. Hardware Verification Test

Because this test sought to evaluate the GPT hardware’s quantitative performance, the identity of the user has no effect on the outcome. Therefore, an experimenter, instead of a consented participant, started near the GPT and walked to the end of the walkway while wearing the GPT harness in each trial.

The participant wore reflective markers bilaterally on the pelvis (iliac crest), hip (greater trochanter), mid-knee line, ankle (lateral malleolus), and toe (5th metatarsal). The Vicon motion capture system recorded 3D marker positions at 100 Hz.

A one-dimensional iLoad Mini force sensor rated up to 222.41 N was fixed to the back of the harness worn by the participant, and the cable was tied to the load side of the sensor via the sensor’s hook. This in-line configuration allowed the sensor to accurately record the cable’s tension during the test. In addition, the remote sensing system was slightly modified so that it generated two signals indicating the states of Ron/Roff and URon/URoff. These signals were recorded, time-stamped, and synced with the marker trajectory data by the Vicon analog-to-digital conversion system at 1000 Hz.

For resistance force control in this test, we used the experimenter’s peak propulsion force magnitude of 140 N as the baseline (BL), and we sequentially tested GPT resistance force values of 0% BL, 20% BL, 30% BL, 40% BL, 50% BL, and 60% BL, with five trials for each force value. The 0% BL condition was included to determine the residual inherent friction from the brake, and the other conditions sought to verify the full functionality of the GPT. Outcome measures in this hardware verification test include statistics about the magnitude of the GPT resistance force and the time delays between the various important events in the system, such as actual and detected heel strike and toe off, as well as resistance force onset and termination.

#### 2.3.2. Pilot Study

The second test served as a pilot study for how effective the GPT is in unilateral propulsion training. In this study, two participants, one healthy male (30 years old) and one female with stroke (50 years old), each repeatedly walked along the overground walkway. The two participants were unfamiliar with the device and were not members of the research team.

Our inclusion criteria for stroke participants were that they needed to be able to walk 10 meters without any assistive device or aid from a therapist. The body mass of the participant with stroke is 97.5 kg, giving a body weight of 956.34 N, and she had injury in her right cortex in March 2016. The left-hand side of her body is paretic. She uses a cane in everyday walking, and she does not wear an ankle-foot orthosis. When she tested the device in the pilot study, the stroke participant walked unassisted, and an occupational therapist (OT) walked next to her, ready to assist should she fall.

The participants wore reflective markers for the Vicon motion-capture system. Note that the OT blocked some markers from correct detected in some trials with the stroke participant, and we removed these trials before reporting results in the next section because we did not have the necessary kinematics data in these trials. In addition, instead of the force sensor in the hardware verification study, we used the embedded in-ground force plates to measure the 3D ground reaction forces, and their locations were unknown to the participants. The participants were instructed to walk naturally at a comfortable pace and to try to maintain the same pace throughout the experiment. At the end of each trial, the stroke participant returned to the GPT by sitting in a wheelchair that was pushed by an experimenter; this time allowed the participant to rest between trials. The healthy participant simply walked back to the starting position.

The conditions for this study are detailed in Table 1 and Table 2. The presentation order was as shown in the tables for each participant. While the healthy participant tested all conditions, the stroke patient could not tolerate greater than 40% BL, due to fatigue. In the supplemental material of this paper, we provide animations that visualize all of the reflective markers’ positions over time for each trial of the pilot study. Anterior/posterior ground reaction force, when available, is drawn as an arrow emerging from the corresponding foot. The arrow’s direction indicates the direction of the forces that the foot is exerting on the ground.

**Figure 7:**
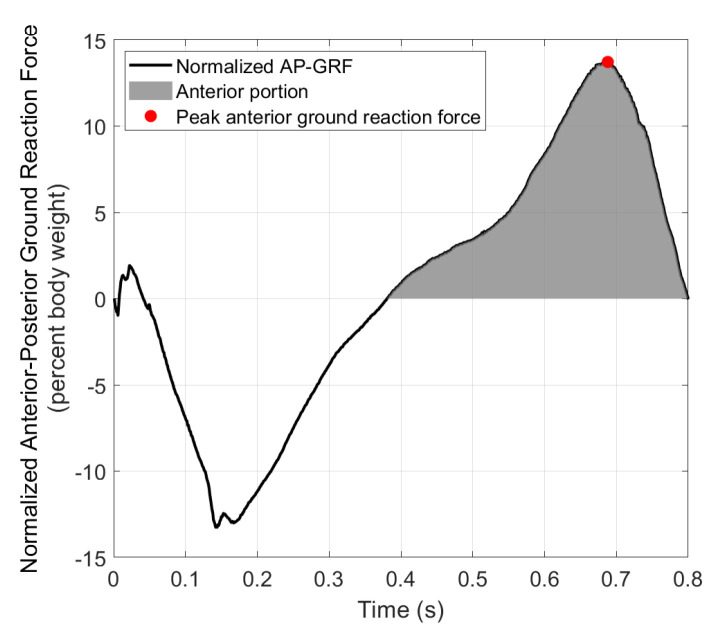
Anterior–posterior ground reaction force from one example step.

Figure 7 shows the anterior–posterior ground reaction force (AP-GRF) normalized by body weight from an example step. AP-GRF in the negative (posterior) direction is the braking force, and AP-GRF in the positive (anterior) direction is the propulsion force, i.e., the reaction force from the participant pushing against the ground to propel the body forward in mid-late stance. Average peak anterior ground reaction forces from the pre-training or the 0% BL condition were used as baselines (BL) to calculate desired GPT resistance for the subsequent conditions for each participant. The BL was 133.3 N for the healthy participant and 19.8 N for the stroke participant.

Secondly, the number of trials in each condition depended on how the participants stepped on the force plates. At the beginning of each trial, the experimenter started the participant in a position that was predicted to result in good force plate steps, which were steps that were squarely on at least one of the force plates and not near the edges. If one or more good force plate steps occurred with a particular limb in a trial, this trial was considered a good trial for that limb. Thus, a trial could be good for both limbs or only one limb, or it could be a bad trial (i.e., no good force plate steps on either side), depending on how the steps were located. Each experiment condition was repeated until at least three good trials for each limb were obtained, and then the next experiment condition was tested. The numbers of total and good trials for each limb are shown in the fourth columns of Table 1 and Table 2. Note that because the stroke participant had relatively short step lengths, some trials yielded more than one good force plate step, as shown in the 30%, and 40% BL conditions in Table 2. Regardless of whether a particular trial is considered ‘good’ for any limb, the participants always walked to the end of the walkway before returning to the GPT and starting another trial. The average number of steps in each trial was 7.3 for the healthy participant and 13.3 for the stroke participant; these average values were obtained from motion-capture data and are thus underestimates of the true numbers of steps, because the motion capture system did not cover the entire walkway.

Outcome measures in the pilot study include peak propulsion forces normalized by body weight, propulsion impulses, step lengths, and walking speeds. The step length of a leg is defined as the distance between the two feet when the leg is leading and at heel strike. For propulsion impulses and step lengths, we normalized the data by the sum of the median data values from the two legs in each test condition. This normalization process eliminates influences from body weight and walking speed. Medians were used instead of mean values to reduce biases from outlier steps. Propulsion impulse and step length asymmetry levels were thus calculated as the difference between the corresponding normalized values of the two legs.

Given the small sample sizes and unmatched data in each condition, we performed the one-tailed nonparametric Fisher–Pitman permutation test for independent samples [35,36] to evaluate performance differences.

## 3. Results

### 3.1. Hardware Verification

**Figure 8:**
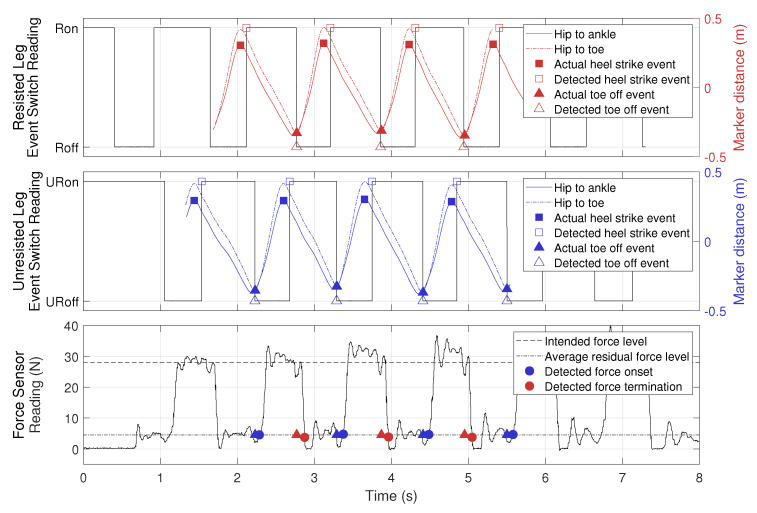
Data from an example trial in the verification test.

Data collected from an example trial are shown in Figure 8. The top panel shows readings from the event switches on the resisted leg, where ‘Ron’ and ‘Roff’ represent the resisted foot being in stance or swing phase, as in Figure 5. Horizontal distances between markers (hip to ankle and hip to toe) were extracted from Vicon trajectory data, where a positive value means that the hip marker is behind the ankle or the toe marker, and vice versa. Therefore, we use the maximum values of hip-to-ankle distance as actual heel strike events and the minimum values of hip-to-toe distance as actual toe off events, an approach validated in [37]. The center panel shows the same data for the unresisted leg. The bottom panel shows force output recorded by the force sensor. We define resistance force onset (blue circles) as when the force value exceeded 4.5 N, which was the average residual force in Table 3. Similarly, resistance force termination (red circles) is defined as when the force value dropped below 4.5 N. The filled blue and red triangles from the top two panels are plotted here at 4.5 N to illustrate the delay in force onset and termination.

Figure 8 shows that as the experimenter walked away from the GPT, data from the event switches beneath the soles of the shoes closely approximated the heel-strike and toe-off events on both legs, following the actual events extracted from the Vicon trajectory data using the coordinate-based algorithm in [37]. In addition, the resistance force behaved as expected, turning on after the unresisted leg entered the swing phase (URoff) and turning off after the resisted leg entered swing phase (Roff). The residual force when the unresisted leg was in the stance phase (after detected force termination and before detected force onset) was non-zero, so the user may still feel some resistance when the brake force is turned off. We believe the residual force is caused by the inertia of the shaft and the part of the brake’s inherent friction that was not canceled by the motor.

To evaluate how fast the GPT reacted to the user’s motion, we calculated time delays between four corresponding pairs of events as listed below (see Figure 8):

- Actual heel strike and detected heel strike;
- Actual toe off and detected toe off;
- Intended resistance force onset and detected resistance force onset;
- Intended resistance force termination and detected resistance force termination.

Thus, the first two types of delays measure how quickly our wireless gait detection system reacts to actual kinematic data, and the last two types of delays measure how fast the overall GPT force control is, including both the wireless system and the brake control. The results are shown in Table 4. Note that the actual and detected toe-off events had a negative delay on average, because the event switches attached to the toe area were not at the very front of the shoe, so they switched from on to off slightly before the actual toe-off event. In the current control scheme, the heel-strike events are detected but not used, so they do not affect GPT performance.

As shown in Table 4, the delays for force termination were slightly larger than those for force onset, because it took some time for tension to release in the cable after the brake was deactivated. Another possible reason for this effect is the hysteresis in the brake’s torque output: when the current through the brake decreases, the output torque may remain at the higher level until the shaft rotates.

Table 3 shows GPT resistance force magnitudes at different levels of intended force output. Since the brake was never activated in the 0% BL condition, we used all data when the participant was walking away from the GPT to calculate the statistics. In the other conditions, we used data when the brake was activated, as illustrated by the segments between the blue and red circles in the bottom panel of Figure 8. The resistance force in the 0% BL condition has a magnitude of 4.5 N, and the GPT resistance force control was more accurate in the 20% BL and 60% BL conditions than in the 30% BL, 40% BL, and 50% BL conditions, where the actual force output was approximately 10% larger than the intended level.

### 3.2. Pilot Study

**Figure 9:**
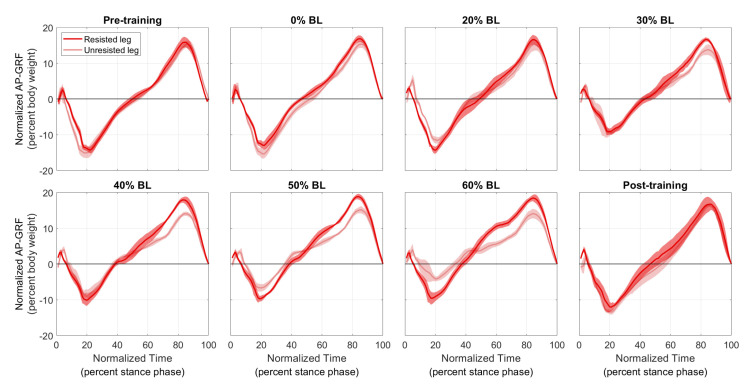
Normalized AP-GRFof the healthy participant. Test conditions are detailed in Table 1. Solid lines represent the normalized mean, and shaded regions represent the standard deviation for each normalized time step.

**Figure 10:**
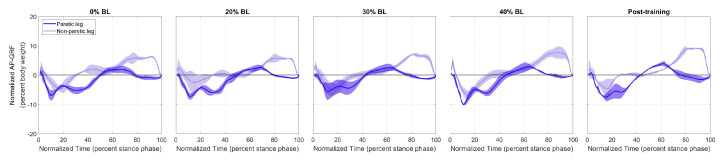
Normalized AP-GRF of the stroke participant. Test conditions are detailed in Table 2.

Figure 9 and Figure 10 show the normalized AP-GRF recorded from the force plates as the two participants walked in each condition. Force magnitude is normalized by each participant’s body weight, and time is normalized by total stance phase time, i.e., 0 for heel strike and 100 for toe off.

**Figure 11:**
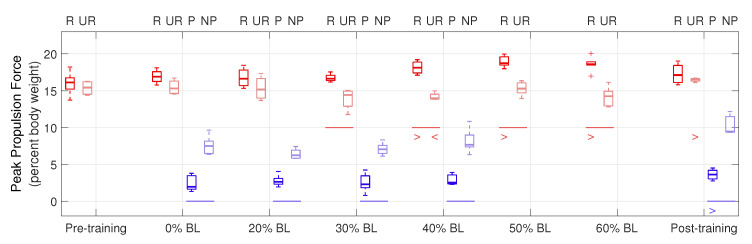
Boxplots of peak propulsion forces in the pilot study.

Peak propulsion forces are shown in Figure 11. ‘R’ and ‘UR’ groups (red boxplots) represent the resisted and unresisted legs of the healthy participant, and ‘P’ and ‘NP’ groups (blue boxplots) represent the paretic and non-paretic legs of the stroke participant. The central lines indicate the median, and the bottom and top edges of the boxes indicate the 25th and 75th percentiles, respectively. A horizontal line below a pair of boxplots shows a significant difference *in the corresponding condition between the two legs* (p<0.05) in the means of data. A ‘>’ or ‘<’ below a boxplot shows a significant increase or decrease in the data mean *of that leg between the corresponding condition and the first tested condition*; for example, the ‘>’ under P in the post-training condition indicates that the paretic leg of the stroke participant generated significantly increased peak propulsion force, compared to the 0% BL condition.

From Figure 11, for the healthy participant, higher GPT resistance led to increased peak propulsion forces by the resisted leg, compared to the unresisted leg (see the red horizontal lines in Figure 11), as shown by the one-tailed nonparametric Fisher–Pitman permutation test under the 30% BL (p=0.014), 40% BL (p<0.001), 50% BL (p=0.004), and 60% BL (p=0.002) conditions. We also performed the one-tailed Fisher–Pitman permutation test for each leg between the pre-training condition and each of the subsequent conditions (see the red ‘>’ and ‘<’ symbols). Significantly increased peak propulsion force was found in the 40% BL (p=0.019), 50% BL (p=0.004), and 60% BL (p=0.029) conditions in the resisted leg. For the unresisted leg, normalized normalized peak propulsion decreased in the 40% BL condition (p=0.044) but increased in the post-training condition (p=0.029). For the stroke participant, the non-paretic leg always generated significantly higher peak propulsion forces than the paretic leg (p<0.028 in all conditions, see the blue horizontal lines). In addition, significantly increased peak propulsion forces were found in the paretic leg in the post-training condition (p=0.048), compared to the 0% BL condition (see the blue ‘>’ symbol).

**Figure 12:**
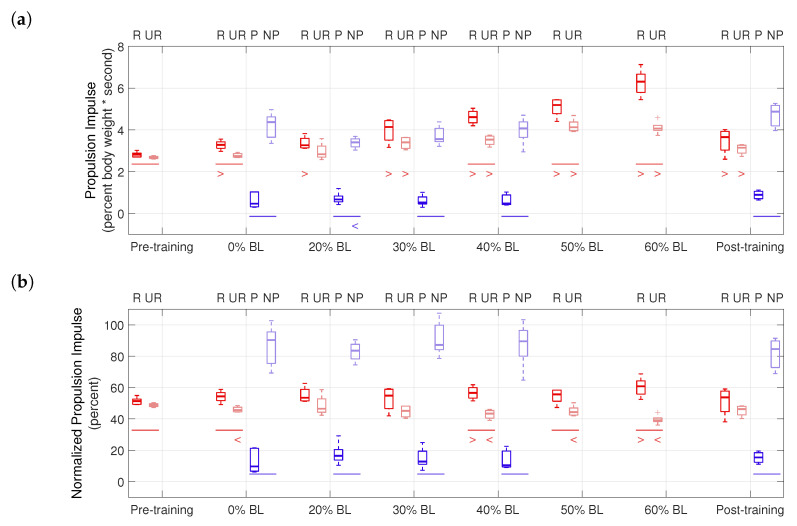
Raw and normalized propulsion impulses in the pilot study. (**a**) Propulsion impulses. (**b**) Propulsion impulses normalized by sum of median values.

Propulsion impulses are shown in Figure 12. For the healthy participant, significant differences between the two legs were found in the pre-training (p=0.038), 0% BL (p<0.001), 40% BL (p=0.014), 50% BL (p=0.008), and 60% BL (p=0.002) conditions. With GPT resistance and in the post-training condition, the healthy participant almost always generated significantly higher propulsion impulses, compared to the pre-training condition (p<0.033 in all cases), except the unresisted leg in the 0% BL and 20% BL conditions. For the stroke participant, the non-paretic side always had significantly higher propulsion impulses (p<0.028 in all conditions). In addition, significantly decreased propulsion impulse was found in the non-paretic leg in the 20% BL condition (p=0.029), compared to the 0% BL condition.

Propulsion impulse asymmetry is shown in Figure 12b and summarized in Table 5. Figure 12b plots raw propulsion impulses from Figure 12a normalized by the sums of median values in each condition, thus illustrating the propulsion impulse contributions of the two legs and eliminating influences of other factors, such as changes in walking speed between different conditions. It can be seen that in the 40% BL and 60% BL conditions, the healthy participant’s normalized propulsion impulse significantly increased for the resisted leg and decreased for the unresisted leg, showing indications of an altered asymmetry level. Table 5 reports the propulsion asymmetry levels as the differences of the median values of the corresponding groups in Figure 12b. Note that because each trial in the pilot study may be good for both limbs or only one limb (see Section 2.3.2), it is infeasible to pair data from steps to show ranges of the asymmetry level in each condition. Thus, Table 5 provides only preliminary insights into the effects of training with the GPT: in the post-training condition, propulsion impulse asymmetry increased by 4.82% compared to the pre-training condition for the healthy participant and reduced by 11.54% compared to the 0% BL condition for the stroke participant.

**Figure 13:**
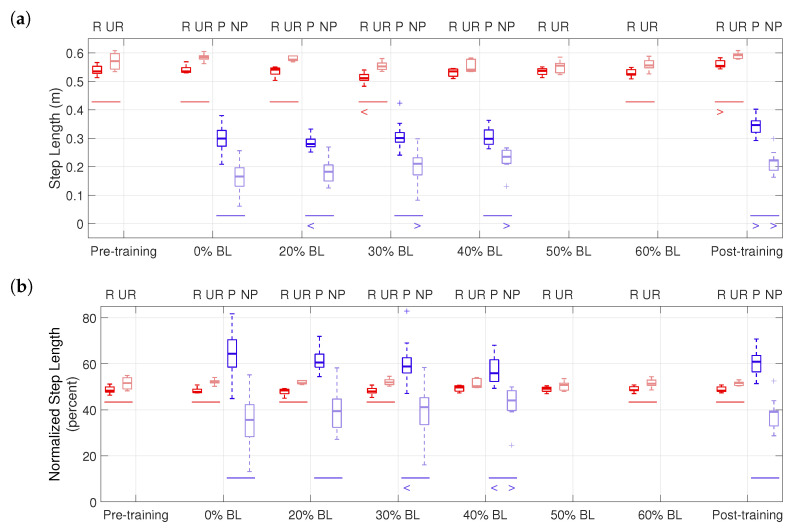
Raw and normalized step lengths in the pilot study. (**a**) Step lengths. (**b**) Step lengths normalized by sum of median values.

Figure 13 shows step lengths in the pilot study. For the healthy participant, significant differences in step lengths between the two legs were found in the pre-training (p=0.013), 0% BL (p=0.001), 20% BL (p=0.004), 30% BL (p<0.001), 60% BL (p=0.004), and post-training (p=0.002) conditions, using the one-tailed nonparametric Fisher–Pitman permutation test, as shown in Figure 13. The step length of the resisted leg significantly decreased in the 30% BL condition (p=0.007) but increased in the post-training condition (p=0.022). For the stroke participant, the paretic side always had significantly higher step lengths (p<0.001 in all conditions). In addition, significantly increased step lengths were found in the non-paretic leg in the 30% BL (p=0.004), 40% BL (p<0.001), and the post-training conditions (p=0.002), compared to the 0% BL condition, while the step lengths of the paretic leg decreased in the 20% BL condition (p=0.045) but increased in the post-training condition (p<0.001).

The step length asymmetry levels are summarized in Table 6. For the stroke participant, the asymmetry level reduced by 6.91% in the post-training condition, compared to the 0% BL condition. In Figure 13b, the stroke participant’s step length significantly increased for the non-paretic leg but decreased for the paretic leg, also suggesting the changed asymmetry level during training.

**Figure 14:**
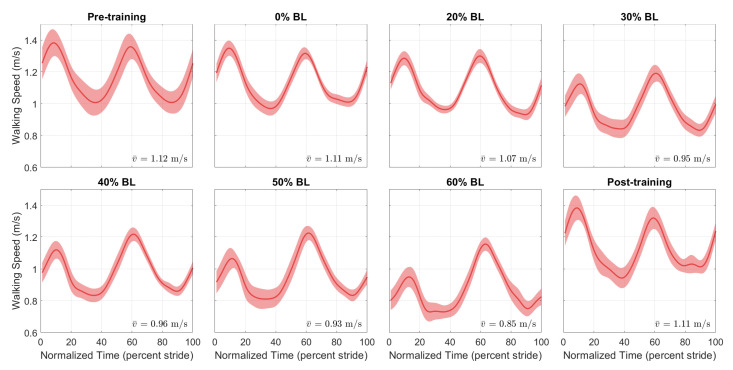
Speed profiles of the healthy participant. Each stride starts at resisted side heel strike (HS) and ends at the next resisted side HS.

**Figure 15:**
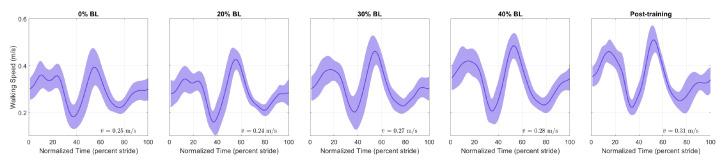
Speed profiles of the stroke survivor participant. Each stride starts at paretic HS and ends at the next paretic HS.

Figure 14 and Figure 15 show the speed profiles normalized by stride time in the pilot study. For the healthy participant, the speed decreased as GPT resistance increased but returned to pre-training levels in the post-training condition. For the stroke participant, walking speed increased with higher GPT resistance, and the increase persisted in the post-training condition.

## 4. Discussion

The presented results demonstrate that the GPT prototype is fully functional. The hardware verification test shows that the GPT reacted quickly to the user’s gait phases and turned the resistance force on and off with correct timing. The magnitudes of the resistance forces were well controlled, as shown by their small standard deviations, but the GPT’s force output should be re-calibrated to make sure the applied resistance matches the intended magnitude. This re-calibration can be easily performed and will not affect any other aspect of the GPT’s function.

In the pilot study, the peak propulsion forces of the healthy participant’s resisted leg significantly increased during training, as we expected. On the other hand, the stroke participant did not show a significant increase during training, possibly because of a lack in muscle strength. Nevertheless, a significant increase in peak propulsion force was found for the paretic leg in the post-training condition, encouraging future studies to evaluate the long-term effect of training with the GPT.

The effectiveness of GPT training is also shown by the propulsion impulse and step length measures. In Figure 12b and Table 5, the healthy participant started with a slight (2.65%) impulse asymmetry, which increased to as much as 21.60% in the 60% BL condition, (i.e., the resisted leg generated as much as 60.80% of total propulsion impulses). In contrast, the stroke participant started with a high level (−80.63%) of propulsion impulse asymmetry, where the paretic leg generated about only 9.69% of total impulses in the 0% BL condition. The highest reduction in impulse asymmetry took place in the 20% BL condition (−67.01%), and the reduction persisted in the post-training condition (−69.09%), with the paretic side contributing about 15.46% of total impulse. Interestingly, the changes in propulsion asymmetry were not monotonous with increasing GPT resistance for either participant, and the healthy participant’s propulsion impulses increased in both legs, which we did not anticipate. Thus determination of the most effective training level and/or the timing of resistance forces may need further experiments. Nonetheless, the GPT was successful in causing greater propulsion impulse contribution from the resisted side for the healthy participant and from the paretic side for the stroke participant, as hypothesized.

As shown in Figure 13b and Table 6, the healthy participant’s step length asymmetry level remained relatively constant throughout the pilot study. For the stroke participant, the step length asymmetry decreased with higher GPT resistance, from 28.81% in the 0% BL condition to as low as 11.87% in the 40% BL condition, and the reduction also persisted in the post-training condition (21.90%). In addition, the step lengths of the non-paretic leg appeared to increase overall (see NP groups in Figure 13a).

The final measure we analyzed is walking speed. As shown in Figure 14, for the healthy participant, there was a reduction in speed throughout the stride, especially with higher GPT resistance values, but the reduction did not persist in the post-training condition. A possible reason is that as the participant pushed harder off the ground under the influence of the GPT, they walked more carefully and slowed down unintentionally, which then may have caused higher propulsion impulses from both legs (Figure 12a). In contrast, the stroke participant walked faster (Figure 15), and the speed increase mostly took place in the 0% to 60% stride region, which contains the period when the paretic leg pushed off the ground and corresponds to the non-paretic step lengths in Figure 13a, which appeared to increase as discussed above. Thus, after only one session, the stroke participant appeared to walk with increased propulsion impulse symmetry and step length symmetry and at faster speeds, suggesting promising effects of training with the GPT.

One side effect to note is that the braking force (negative AP-GRF) seemed to decrease, especially for the healthy participant (Figure 9). Since the current control scheme applies resistance until a short period of time after the resisted side toe-off, the brake also helps with braking on the unresisted side. In addition, this resistance force present during single support of the unresisted side may also explain the increase in propulsion impulses from the unresisted leg of the healthy participant in Figure 12a. The reduction in braking force was not as apparent for the stroke participant in Figure 10, perhaps because they walked slowly. Nonetheless, neither participant seemed to experience any discomfort or danger because of the reduced braking force in our pilot study.

Compared to other training schemes that target stance-phase propulsion, such as applying torque pulses to joints [27] and accelerating the belt of the trailing limb [28], the GPT enables a different intervention by applying resistance forces at the pelvis, and it allows over-ground walking as opposed to relying on a treadmill. Nevertheless, our results agree with previous research that stance-phase propulsion is a promising area for gait rehabilitation, and more research would be beneficial to understanding the efficacy of these approaches.

It is important to note that this pilot study was performed with only one healthy young adult and only one stroke survivor. Since we were conducting preliminary testing with only two participants, we did not apply the common practice of randomizing the presentation order, as we were worried that any order effects would interfere with our ability to understand how the device affects walking. We also wanted participants to gain comfort with the device and perform many steps in each condition. Thus, we ordered the trials so that the resistance increased over the course of the study. Moreover, additional evaluation with more participants could obtain more evidence regarding increased propulsion impulse from the resisted/paretic leg as well as the relationship between GPT resistance and the resultant impulse propulsion asymmetry. Collecting data from more people with stroke would be particularly valuable because of the variety of ways this pathology manifests in the locomotor system. Finally, more data are needed to determine whether changing the timing of the force control could achieve a better training effect.

## 5. Conclusions

In conclusion, we designed and evaluated a gait rehabilitation device that applies resistance forces during stance phase. The GPT does not rely on a treadmill and enables training while walking overground, which may lower the total cost, but more space may be required for users to walk a longer distance in each trial. Additional studies with more participants are needed to determine whether there are predictable relationships between GPT resistance and propulsion asymmetry and whether sustained practice with GPT resistance targeting the weaker leg for people with stroke would lead to improved propulsion impulse symmetry. Nonetheless, the pilot study shows the promising effects of training with the GPT.

## 6. Patents

The work reported in this manuscript resulted in patent Gait Rehabilitation Systems, Methods, and Apparatuses Thereof, US Patent No. US 10456318 B2.

## Figures and Tables

**Table 1:** Tested conditions with the healthy participant in the pilot study, BL = 133.3 N. For number of trials, the letter designations T, R, and UR represent the numbers of total, good for resisted leg, and good for unresisted leg trials.

Condition	Participant Wearing GPT	Intended GPT Resistance Level	Number of Trials
Pre-training	No	Not applicable	6T 6R 4UR
0% BL	Yes	0 N	5T 4R 4UR
20% BL	Yes	26.7 N	5T 4R 4UR
30% BL	Yes	40.0 N	7T 4R 4UR
40% BL	Yes	53.3 N	5T 4R 4UR
50% BL	Yes	66.7 N	5T 5R 5UR
60% BL	Yes	93.3 N	6T 6R 5UR
Post-training	Yes	0 N	7T 4R 4UR

**Table 2:** Tested conditions with the stroke participant in the pilot study, BL = 19.8 N.

Condition	Participant Wearing GPT	Intended GPT Resistance Level	Number of Trials
0% BL	Yes	0 N	12T 5R 5UR
20% BL	Yes	4.0 N	6T 5R 5UR
30% BL	Yes	6.0 N	7T 5R 11UR
40% BL	Yes	8.0 N	5T 3R 8UR
Post-training	Yes	0 N	5T 4R 3UR

**Table 3:** Force magnitudes in the hardware verification test, BL = 140 N.

Condition	Intended GPT Resistance Level	Actual Force Output (μ±σ)
0% BL	0 N	4.5 N±1.1 N
20% BL	28 N	29.6 N±2.8 N
30% BL	42 N	47.5 N±3.7 N
40% BL	56 N	62.5 N±3.8 N
50% BL	70 N	77.7 N±3.8 N
60% BL	84 N	85.6 N±4.1 N

**Table 4:** Time delays between corresponding events in the hardware verification test.

Event	Time Delay (μ±σ)
Actual and detected heel strike	0.082 s±0.019 s
Actual and detected toe off	−0.005 s±0.013 s
Intended and detected force onset	0.051 s±0.022 s
Intended and detected force termination	0.113 s±0.020 s

**Table 5:** Propulsion impulse asymmetry. A positive asymmetry means that the impulse generated by the resisted or paretic leg is higher. DNT stands for ‘Did Not Test’ for the stroke participant.

Condition	Propulsion Impulse Asymmetry (Percent)	
	Healthy Participant	Stroke Participant
Pre-training	2.65	DNT
0% BL	8.80	−80.63
20% BL	6.98	−67.01
30% BL	9.74	−74.43
40% BL	13.20	−78.97
50% BL	11.39	DNT
60% BL	21.60	DNT
Post-training	7.47	−69.09

**Table 6:** Step length asymmetry. A positive asymmetry means step length of the resisted or paretic leg is longer.

Condition	Step Length Asymmetry (Percent)	
	Healthy Participant	Stroke Participant
Pre-training	−3.24	DNT
0% BL	−4.42	28.81
20% BL	−3.11	21.20
30% BL	−3.91	17.67
40% BL	−0.58	11.87
50% BL	−1.70	DNT
60% BL	−2.81	DNT
Post-training	−3.29	21.90

## Data Availability

The data presented in this study are available in the Supplementary Materials.

## Supplementary Materials

The following are available online at https://www.mdpi.com/article/10.3390/s21196617/s1.
